# Relationship between body mass index and residential segregation in large cities of Latin America

**DOI:** 10.1186/s12889-024-19074-9

**Published:** 2024-06-22

**Authors:** Tamara Doberti Herrera, Lorena Rodríguez Osiac, Sandra Flores-Alvarado, Carolina Pérez Ferrer, Diana Higuera, Leticia de Oliveira Cardoso

**Affiliations:** 1 Escuela de Salud Pública, Universidad de Chile, Santiago, Chile; 2 CONACYT-National Institute of Public Health, Mexico DF, Mexico; 3 School of Medicine, Universidad de los Andes, Bogotá, Colombia; 4 Oswaldo Cruz Foundation, Rio de Janeiro, Brazil

**Keywords:** Residential segregation, Obesity, Latin America

## Abstract

**Background:**

Obesity is a global health problem, and its connection with social and environmental factors is well-established. Social factors, such as urban segregation, may impact obesity through various mechanisms, including food and physical activity environments, as well as social norms and networks. This multilevel study aims to examine the effect of socio-economic residential segregation of Latin American cities on the obesity of individuals within those cities.

**Methods:**

We analyzed data from national surveys for a total of 59,340 individuals of 18–70 years of age, conducted in 156 cities across Brazil, Chile, Colombia, and Mexico between 2007 and 2013. We adjusted two-level linear mixed models for body mass index (BMI) stratified by sex and country, controlling for age, educational level and poverty. Separate models were built for dissimilarity and isolation segregation indices.

**Results:**

The relationships between segregation indices and BMI were mostly not statistically significant, and in some cases, they were opposite to what was expected. The only significant relationships were observed in Colombian men, using the dissimilarity index (-7.5 [95% CI: -14.4, -0.5]) and in Colombian women, using the isolation index (-7.9 [95% CI: -14.1, -1.7]).

**Conclusions:**

While individual-level factors cannot fully explain differences among people in the same city, segregation indices may help. However, we found that in some cases, the relationship between BMI and segregation indices is opposite to what is expected based on prior literature. This should be considered in examining the phenomenon. Further research on obesogenic environments in segregated neighborhoods could provide valuable evidence.

**Supplementary Information:**

The online version contains supplementary material available at 10.1186/s12889-024-19074-9.

## Background

Obesity is a global health problem, not only because of its associated health complications, but also because of its high economic and social cost [1]. The World Health Organization (WHO) reports that from 1975 to the present obesity has tripled, affecting about 2 billion worldwide. By 2016, 39% of people over 18 worldwide were overweight and 13% obese [2]. The Food and Agriculture Organization of the United Nations (FAO) and the Pan American Health Organization (PAHO) state in their 2016 Food and Nutrition Security Landscape Report that 58% of Latin America and the Caribbean inhabitants are overweight and 23% are obese, with Chile, Mexico and Bahamas presenting the highest national prevalence [3].

Obesity is caused by a number of structural, environmental and individual factors. Among these are social determinants. According to WHO, social determinants are *"the circumstances into which people are born, grow, work, live and age, including the broader set of forces and systems that influence the conditions of everyday life"* [4]. This model attempts to explain how health is affected by the interaction between social determinants and individual factors. There are two levels of determinants: 1) Structural, which influence social stratification and define the socioeconomic position of the individual, such as gender, ethnicity, race, political context; 2) Intermediate, which determine differences in exposure and vulnerability to health-detrimental conditions, including material conditions (e.g. housing, characteristics of the built environment), biological factors such as nutrition or lifestyles, among other factors [5, 6]. Therefore, the social determinants of health act at the individual level through intermediate determinants, but also at the population level through structural determinants and their interaction with intermediate determinants, causing inequalities and inequity in health [4].

The most consistent upstream social determinant of obesity is socio-economic status and inequality. In higher-income countries, lower socio-economic status—and inequality in general—is associated with higher levels of obesity in adult [7]. In Europa [8, 9] and Latin America, obesity in children under 11 is linked to lower socio-economic status and poor environmental conditions, with more obesity where socio-economic deprivation is greater [10–12]. This may occur because families lack adequate access to healthcare and opportunities for healthy behaviours, consequently increasing the incidence of childhood overweight due to poor nutrition [8, 13]. This effect manifests not only at the individual level but also at the population level, shaped by urban segregation and environmental characteristics.

Urban segregation impacts in the socio-cultural or social environment (i.e., the way we relate to each other), something that has been studied extensively. The characteristics of other people, such as family members, friends, colleagues, neighbors, and other "social network" members, importantly correlate with obesity risk. Christakis et al. have shown that obesity spreads through social networks over time, mainly via siblings and friends. [14] Some explanations of the perceived effect of social networks include social contagion (whereby the network influences obesity-related behaviors), social capital (whereby a sense of belonging and social support influence obesity-related behaviors), and social selection (whereby an individual's network is a function of similarity of their weight) [15]. A recent systematic review showed that the strongest social environmental correlates of obesity were social capital and collective efficacy, however, in general, few social environmental factors were consistently related to adult obesity [16].

Other important social determinant of health is urban residential segregation, which refers to the different territorial distribution of individuals belonging to a social group, compared to those belonging to another social group [17–19]. According to Sabatini, residential segregation can be defined "*as the degree of spatial proximity or territorial agglomeration of families belonging to the same social group, whether it is defined in ethnic, age, religious or socioeconomic preferences, among other possibilities*" [19]. Massey and Denton define five dimensions to evaluate residential segregation, the most used are equality and exposure, measured by the dissimilarity and the isolation indices [17].

Some authors have studied race, ethnic, or socioeconomic residential segregation in Latin America, particularly in Mexico and Chile, concluding that the sectors with the lowest income and socioeconomic level are more segregated, and that larger are cities are more segregated than smaller ones [20–22]. The same is observed in Colombia and Brazil, where racial residential segregation is also highly present [23, 24]. The main factors that influence segregation in Latin America are globalization, market deregulation, market diversification in land use, high degree of insecurity in cities, and the desire for exclusivity of emerging groups [23–26].

Residential segregation is measured by segregation indices that compare characteristic of individuals (e.g., religion, race, SES) living in a subarea (e.g., neighborhoods) to those of the overall area (e.g., cities) [15, 27]. The indices that are more frequently used in health studies, particularly in health and obesity research, are the dissimilarity index, which measures equality; and isolation index, which measures the interaction between social groups [28–34]. Residential segregation may have an impact on obesity by shaping the availability of resources, access to and promotion of healthy or unhealthy foods, as well as physical activity resources. Similarly, the quality and features of homes, infrastructure, and neighborhood facilities can influence behaviors and subsequent health outcomes [7].

Diet-related non-communicable diseases, including obesity, are affected by environmental, social conditions and some social determinants [35]. Contexts (environments) that promote less healthy eating are called obesogenic environments. These are characterized by a high supply of ultra-processed food, low availability of fruits, vegetables and other healthy foods, and powerful marketing strategies for less healthy foods [36]. The built environment is a component of the obesogenic environment defined as the community’s global structure for the physical context, including green areas availability, "walkability", and sunlight access [37], and influences the risk of obesity through the area of residence, income, time watching TV, environment "walkability", land use, urban expansion, and level of deprivation [38].

Residential segregation has been linked to obesogenic environments that promote unhealthy diets. Various studies show that residential segregation affects diet and exercise, concluding that disparities between neighborhoods create disparities both in nutritional and health status [39–42]. For instance, the distribution of supermarkets, small markets, and food stores is related to the environment's socio-economical level, which influences the diversity of available food types [43]. A 2002 study in the United States found more supermarkets in the wealthiest neighborhoods, and three times less alcohol spending stores than in the poorest ones [44]. Pool and collaborators in 2018 conducted a longitudinal study of the relationship between exposure to obesogenic environments and race residential segregation. They found that black women living in highly segregated neighborhoods were 30% more likely to become obese than black women living in neighborhoods with low levels of segregation [45]. In relation to gender, according to Robinovich et al., the low SES of the neighborhood is associated with greater obesity in women in Chile. [46] In relation to race, in 2012 Corral and collaborators found that the probabilities of being obese for the Afroamerican population living in highly segregated areas were 26.5% higher than for those living in areas with low residential segregation index [39, 47].

Previous studies in the USA indicate that there is an association between residential segregation and BMI, with changes in neighborhood racial composition and economic resources being strong predictors of the differences, posing as challenges that it is necessary to continue searching for mediators between segregation and BMI in order to plan effective policies [48]. Few studies have examined the relationship between segregation and obesity in the large growing cities of Latin America, one of the most urbanized regions in the world. The literature in this Region has focused primarily on relating individual socioeconomic conditions to nutritional status, not necessarily on how the grouping of people influences malnutrition.

In this context, the aim of this study was to examine the effect of socio-economic segregation of Latin American cities on the obesity of individuals within those cities. Recognizing the gender disparities highlighted by previous research, our research specifically aims to investigate how socio-economic segregation affects obesity rates differently between men and women in these urban environments. By considering 156 cities in Brazil, Chile, Colombia, and Mexico, and employing harmonized survey data from the SALURBAL (Urban Health in Latin America), we conducted a multilevel analysis that integrates a gender perspective to better understand the socio-economic determinants of obesity.

## Methods

### Data

We used a harmonized dataset built by the SALURBAL project [49], which contains information for ${n}_{i}=\text{59,340}$ individuals of both sexes with ages between 18 and 70 years nested in ${n}_{j}=156$ cities in four Latin American countries: Brazil, Chile, Colombia, and Mexico. Information is recorded both at the individual and city levels (Table 1). City units were defined by SALURBAL as urban agglomerations of at least 100,000 residents in 2010 defined by a collection of adjacent municipalities (or similar) that are part of the apparent urban extent.

**Table 1:** Variable description and sources

Level and subindexes	Source		Variable name	Mean (SD) [Min, Max] / %
Individual i = 1, …, 59,340	**Health surveys**	PNS 2013 – Brazil ENS 2010 – Chile ENSI 2007 – Colombia ENSANUT 2012 – Mexico	BMI^a^	27.5 (5.3) [18.5, 67.4]
			Education^b^	None: 18.6% Primary: 35.3% Secondary: 33.8% Tertiary: 12.4%
			Age	[18,31]: 31.4% (31,44]: 32.0% (44,57]: 22.7% (57,70]: 13.8%
			Sex	Female: 57.7% Male: 42.3%
City j = 1, …, 156	**Census**	2010 – Brazil 2002 – Chile 2005 – Colombia 2010 – Mexico	Dissimilarity	0.264 (0.055) [0.113, 0.409]
			Isolation	0.225 (0.131) [0.085, 0.594]
	**Socioeconomic surveys**	PNAD 2010 – Brazil CASEN 2015 – Chile GEIH 2010 – Colombia ENIGH 2010 – Mexico	Poverty^c^	0.376 (0.159) [0.044, 0.711]

*SD* Standard Deviation, *Min* Minimum, *Max* Maximum, *%* Percentage, *BMI* Body Mass Index, *PNS* Pesquisa Nacional de Saúde, *ENS* Encuesta Nacional de Salud, *ENSIN* Encuesta Nacional de la Situación Nutricional, *ENSANUT* Encuesta Nacional de Salud y Nutrición, *PNAD* Pesquisa Nacional por Amostra de Domicilios, *CASEN* Encuesta de Caracterización Socioeconómica Nacional, *GEIH* Gran encuesta integrada de hogares, *ENIGH* Encuesta Nacional de Ingresos y Gastos de los Hogares

^a﻿^BMI was calculated from measured weight and height in all surveys

^b﻿^Education was the highest education level completed by respondent of the National Health Survey of each country

^c﻿^Poverty was the proportion of the population in the defined geographic area living in households with household income below the national income poverty line (from census data of each country)

The individual-level variables were collected from governmental national health surveys, and they include BMI, sex, age, and educational level. At the city level, the dataset includes the SES residential segregation indices of dissimilarity (equality dimension) and isolation (exposure dimension) constructed from census information for smaller areas (akin to census tracts or neighborhoods) in each city [17, 19]. We derived two sets of segregation indices based on SES proxies: one based on education, defined by the proportion of people over 25 years old (15 years old for Mexico for further details, see the Supplementary material) who have completed primary education, and one bases on income defined by the proportion of people living with ≤ 2 minimum wages. At the city level we also included the percentage of people living below the poverty line based on governmental socio-economic surveys performed by each country. Only cities and individuals with complete information for all the variables were included in the analyses. For more details regarding the surveys and variables, see Table 1.

### Construction of segregation indices

There are five theoretical dimensions to evaluate residential segregation. Equality (also known as evenness), exposure, concentration, centralization, and agglomeration [18]. The dimensions are independent from each other, each of them can be measure by different segregation indices. The first two dimensions are the most used in the study of the effects of residential segregation in health, and the most used indices are the dissimilarity index and the isolation index, respectively [18, 28, 50]. The dissimilarity index represents the proportion of a group’s population that would have to change residence to achieve an even distribution across the city area overall, while the isolation index measures the probably that members of a social group share neighborhoods with only members of the same social group. Both indices contribute to a better understanding of inequalities.

#### Dissimilarity index

The dissimilarity index is a measure of equality or evenness of a social group in a geographic area, and captures the proportion of a group’s population, which in this case corresponds to people who have not completed primary education, that would have to change residence to achieve an even distribution [51]. The index ranges from 0 (complete integration) to 1 (complete segregation) [45]. It is calculated as:

$$dissimilarit{y}_{j}=\frac{1}{2}{\sum }_{k=1}^{{n}_{k}}\left|\frac{{a}_{k}}{{A}_{j}}-\frac{{b}_{k}}{{B}_{j}}\right|$$

Where ${n}_{k}$ is the number of neighborhoods units, ${a}_{k}$ is the number individuals of group A in the k^th^ unit, ${A}_{j}$ is the total number of group A in the j^th^ city area, ${b}_{K}$ is the total number of group B in the k^th^ unit, and ${B}_{j}$ is the total number of group B in the city area. The city unit consists of urban agglomerations formed by a collection of adjacent municipalities and the neighborhood units consist of census tract, as defined by each country’s census.

For Brazil, the index is constructed based on household income, Group A includes individuals with household income $\le$ 2 times the minimum wage; while Group B include those with household income $>$ 2 times minimum wage. For Chile, Colombia, and Mexico it is based on completion of primary education by the population over 25 years old (15 years old for Mexico): Group A contains individuals with incomplete primary education and Group B, those with completed primary education.

#### Isolation index

The isolation index measures the extent to which a social group lives in neighborhoods where they are exposed only to other members of the same social group, in this case, people with the same educational level. It is computed as a weighted average of the proportion of the social group in each area [51]. The values range from 0 to 1, where a score near 0 indicates that the social group is completely integrated with the other social groups, and 1 means the social group is completely isolated from others, that is, more segregated. It is calculated as:

$${isolation}_{j}={\sum }_{k=1}^{{n}_{k}}\frac{{b}_{k}}{{B}_{j}}\cdot \frac{{b}_{k}}{{t}_{k}}$$

Where ${b}_{k}$ is the number of group B in the k^th^ sub-city unit, ${B}_{j}$ is the total number of group B in the j^th^ city area, and ${t}_{k}$ is the total population in the k^th^ unit. The city unit is defined analogously to the dissimilarity index.

For Brazil, the index is constructed based on household income, ≤ 2 minimum wage vs. others. For Chile, Colombia, and Mexico it is based on the proportion of the population under 25 years old (15 for Mexico) with incomplete primary education. Mexico’s cut-off age was lower for the same reason stated for dissimilarity index construction.

### Statistical analysis

We conducted an exploratory analysis of the distribution of the variables stratifying by sex and country. The response variable of interest is the individual BMI within the cities, and the exposure variables are both segregation indices at the city level (Table 1). Adjustment variables were defined at the individual and city levels (Table 1). Since city economic conditions may influence both BMI and segregation indices [20, 25, 52, 53], we controlled for the city's poverty and for individual education level. Since BMI is correlated with age [47], we adjusted for age using age categories to capture any non-linearities. To account for the possible correlation between individuals from the same city and the exposure variables, we used two-level linear mixed models of continuous response. These models allow us to partition the variance components in two: city variance and individual within city variance. Also, due to the different effects that residential segregation has on individual of each gender [46, 54], and the different stages of nutritional transition in each country [55–57], we stratified by sex (proxy of gender) and by country with a random intercept for each city. Additionally, we separately modeled each segregation index because they measure two dimensions of the same construct, possibly leading to collinearity issues if mutually adjusted [19]. To analyze the variance components, we used a null model for each of the sex and country strata, which only included the individual response variable nested in the cities. Additionally, three analysis models were fitted for each segregation index, also stratified by sex and country [58]. The first model included only residential segregation; the second included segregation and the adjustment covariates at the individual level, age, and education; the third was the complete model and it included segregation and all covariates at the city and individual level. The complete model can be described as:

$$BM{I}_{ji}=[{\beta }_{0}+{\beta }_{0j}]+{\beta }_{1}Segregatio{n}_{j}+{\beta }_{2}Povert{y}_{j}+{\beta }_{3}Ag{e}_{ji }+{\beta }_{4}Educatio{n}_{ji}+{\epsilon }_{ji}$$

Where segregation corresponds to the dissimilarity or isolation index; ${\beta }_{0}$ represents the common intercept and ${\beta }_{0j}$ represents the city-specific intercepts; ${\beta }_{1}$ and ${\beta }_{2}$ represent the coefficients for each of the variables at the city level; ${\beta }_{3}$ and ${\beta }_{4}$ represent the coefficients for the variables at the individual level; ${\epsilon }_{ji}\sim Normal(0,{{\sigma }^{2}}_{e})$ corresponds to the random errors of the model and ${{\sigma }^{2}}_{e}$ to the variance within cities; ${\beta }_{0j}\sim Normal(0,{{\sigma }^{2}}_{j})$ corresponds to the random component due to variability among cities, while ${{\sigma }^{2}}_{j}$ is the between cities variance. The goodness of fit of the models was compared using the Akaike and Bayesian information criterion (AIC and BIC). We carried out a secondary analysis to evaluate the possible effect modification of SES adjustment variables on segregation indices by adjusting two new versions of the fully adjusted model, adding an interaction between the segregation variable and the SES adjustment variable (poverty at city level and education at individual level). We based our interpretations on the fully adjusted model.

All statistical analyzes were performed in R [59]. All methods and procedures carried out in the study were performed in accordance with relevant guidelines and regulations and the results were reported following the LEVEL guide [58].

## Results

The final dataset consisted of 59,340 individuals (34,254 women and 25,086 men) grouped in 157 cities in Brazil (*n* = 27), Chile (*n* = 22), Colombia (*n* = 17), and Mexico (*n* = 91). Table 2 shows distributions of city and individual-level variables by country. The dissimilarity index had the highest value in Brazil and the lowest in Chile. The isolation index had the highest value in Brazil and the lowest in Mexico. City poverty was highest in Mexico and lowest in Chile.

**Table 2:** Variable description by country and sex

		**Brazil**		**Chile**		**Colombia**		**Mexico**		**Overall**	
**City level**		(*n* = 27)		(*n* = 22)		(*n* = 17)		(*n* = 91)		(*n* = 157)	
	**Dissimilarity**										
	Mean (SD)	0.33 (0.041)		0.25 (0.045)		0.27 (0.051)		0.25 (0.050)		0.26 (0.055)	
	Median [Min, Max]	0.32 [0.26, 0.41]		0.23 [0.17, 0.34]		0.28 [0.19, 0.41]		0.24 [0.11, 0.34]		0.27 [0.11, 0.41]	
	**Isolation**										
	Mean (SD)	0.45 (0.10)		0.21 (0.059)		0.33 (0.064)		0.14 (0.031)		0.22 (0.13)	
	Median [Min, Max]	0.44 [0.21, 0.59]		0.22 [0.13, 0.32]		0.33 [0.23, 0.47]		0.14 [0.085, 0.22]		0.16 [0.085, 0.59]	
	**Poverty**										
	Mean (SD)	0.30 (0.10)		0.11 (0.045)		0.35 (0.11)		0.47 (0.098)		0.38 (0.16)	
	Median [Min, Max]	0.35 [0.088, 0.43]		0.10 [0.044, 0.20]		0.39 [0.11, 0.53]		0.46 [0.28, 0.71]		0.40 [0.044, 0.71]	
**Individual level**		**Female**	**Male**	**Female**	**Male**	**Female**	**Male**	**Female**	**Male**	**Female**	**Male**
		(*n* = 18,547)	(*n* = 13,892)	(*n* = 1492)	(*n* = 1026)	(*n* = 1986)	(*n* = 1327)	(*n* = 12,229)	(*n* = 8841)	(*n* = 34,254)	(*n* = 25,086)
	**BMI**										
	Mean (SD)	27.1 (5.32)	26.6 (4.51)	28.1 (5.25)	27.7 (4.27)	26.0 (4.89)	25.1 (4.26)	29.2 (5.93)	27.9 (4.94)	27.8 (5.62)	27.0 (4.71)
	Median [Min, Max]	26.2 [18.5, 64.6]	26.0 [18.5, 66.4]	27.3 [18.6, 48.1]	27.4 [18.7, 53.0]	25.0 [18.5, 58.3]	24.4 [18.5, 61.2]	28.5 [18.5, 67.4]	27.5 [18.5, 62.2]	26.9 [18.5, 67.4]	26.5 [18.5, 66.4]
	**Age**										
	[18,31]	29.20%	31.20%	24.50%	25.10%	35.00%	37.30%	32.20%	35.20%	30.40%	32.70%
	(31,44]	32.60%	31.60%	27.10%	29.30%	35.80%	32.20%	33.50%	29.90%	32.80%	30.90%
	(44,57]	23.00%	23.50%	27.90%	25.90%	17.70%	18.40%	22.30%	22.10%	22.70%	22.80%
	(57,70]	15.20%	13.70%	20.50%	19.60%	11.50%	12.10%	11.90%	12.80%	14.10%	13.50%
	**Education**										
	None	21.00%	20.90%	9.50%	4.90%	18.10%	16.60%	17.70%	14.80%	19.10%	17.80%
	Primary	23.50%	25.10%	33.00%	33.10%	34.00%	37.20%	52.90%	52.30%	35.00%	35.60%
	Secondary	39.70%	39.00%	47.70%	53.40%	39.90%	38.30%	22.10%	22.70%	33.80%	33.80%
	Tertiary	15.80%	15.10%	9.90%	8.60%	8.00%	8.00%	7.40%	10.20%	12.10%	12.70%

*N* sample size, *SD* Standard Deviation, *Min* Minimum, *Max* Maximum, *BMI* Body Mass Index

**Fig. 1:**
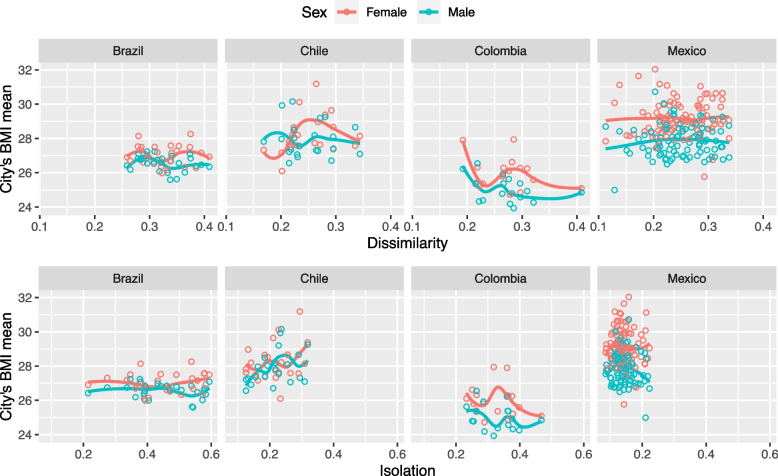
City's body mass index distribution by sex and country for the explanatory variables in the study. BMI = Body Mass Index. Points represent cities and lines represent loess smoothers of the relationship between segregation indices and cities mean BMI. Red lines and points represent females and blue lines represent males

Mean BMI was highest in Chile and Mexico and lowest in Colombia. Higher values were observed for women than men. The Chile survey sample tended towards older and more educated individuals compared to that from other countries. At the individual level, we observed that BMI increases with age for both sexes, however, in men it stabilizes at a younger age than in women; on the other hand, for women in Mexico and Brazil the BMI decreased when education increased, while the opposite occurred with men (Fig. S1). Fig. 1 displays BMI by segregation levels, stratified by country. Associations were not consistent between countries. BMI increased with higher segregation in Chile but decreased with higher segregation in Colombia. No clear associations were observed in Mexico or Brazil.

Table 3 shows mean differences in BMI by selected city and individual-level characteristics. BMI was generally higher in older than in younger ages. Higher education was associated with lower BMI in women, but the opposite pattern was observed in most countries in men (Fig. S1). There were no consistent associations of the segregation indices with BMI: positive associations were observed in some countries and inverse association in others, with most of them not being statistically significant. Intraclass correlation coefficients (ICC) in the fully adjusted models (Tables 3 and 4) ranged from 0.000 to 0.018, showing that most of the variability was within cities.

**Table 3:** Mean differences in BMI associated with city and individual-level variables in fully adjusted model

	**Females mean differences in BMI (95% CI)**				**Males mean differences in BMI (95% CI)**			
	**Brazil**	**Chile**	**Colombia**	**Mexico**	**Brazil**	**Chile**	**Colombia**	**Mexico**
City level variables								
Dissimilarity^a^	-2.3 (-7.5—2.8)	5.0 (-6.0—16.0)	-6.5 (-13.7—0.8)	-1.6 (-6.1—2.9)	-3.6 (-7.9—0.8)	-2.3 (-9.5—4.9)	**-7.5 (-14.4—-0.5)**	-2.0 (-6.3—2.2)
Poverty^b^	0.8 (-1.2—2.8)	-1.7 (-12.8—9.4)	3.3 (0.3—6.3)	-3.5 (-5.8—-1.3)	0.6 (-1.9—2.3)	4. (-3.2—12.1)	1.6 (-1.2—4.4)	-1.8 (-4.0—0.4)
Individual level variables								
Age[18,30]	Ref	Ref	Ref	Ref	Ref	Ref	Ref	Ref
Age(31,44]	1.9 (1.7—2.1)	1.5 (0.8—2.2)	2.1 (1.6—2.6)	2.7 (2.5—3.0)	1.9 (1.7—2.1)	1.5 (0.8—2.2)	2.2 (1.7—2.8)	2.3 (2.1—2.6)
Age(44,57]	2.4 (2.2—2.7)	2.3 (1.6—3.1)	4.0 (3.4—4.6)	3.5 (3.2—3.8)	2.2 (2.0—2.4)	2.1 (1.4—2.8)	1.8 (1.1—2.4)	2.4 (2.1—2.7)
Age(57,70]	2.4 (2.2—2.7)	2.8 (2.0—3.6)	3.6 (2.8—4.3)	3.2 (2.8—3.6)	2.1 (1.9—2.4)	2.1 (1.3—2.9)	1.9 (1.1—2.7)	1.8 (1.5—2.2)
Education None	Ref	Ref	Ref	Ref	Ref	Ref	Ref	Ref
Education Primary	-0.2 (-0.4—0.1)	-0.2 (-1.2—0.8)	-0.4 (-1.0—0.2)	-0.7 (-0.5—0.1)	0.2 (-0.0—0.4)	0.2 (-1.1—1.5)	0.4 (-0.3—1.0)	0.5 (0.2- 0.8)
Education Secondary	-1.0 (-1.2—-0.8)	-1.5 (-2.5—-0.5)	-0.8 (-1.4—-0.2)	-1.0 (-1.4—-0.7)	1.0 (0.8—1.2)	0.1 (-1.2—1.4)	0.5 (-0.2—1.2)	0.7 (0.4—1.1)
Education Tertiary	-1.9 (-2.2—-1.6)	-2.2 (-3.4—-1.0)	-1.0 (-1.9—-0.2)	-1.9 (-2.3—-1.4)	0.9 (0.7—1.2)	-0.8 (-2.3—0.7)	1.2 (0.2—2.2)	1.2 (0.8—1.7)
**Random effects**	Variance	Variance	Variance	Variance	Variance	Variance	Variance	Variance
Between city	0.179	0.372	0.234	0.496	0.115	0.010	0.119	0.417
Within city	26.507	24.983	20.755	31.925	19.241	17.407	17.003	22.721
ICC	0.007	0.015	0.011	0.015	0.006	0.001	0.007	0.018

Results for intermediate models and models without stratification are available in Tables S2 and S3. In bold: confidence intervals that exclude 0, indicating statistically significant coefficients for the explanatory variable

*P*-values are available in Table S4

^a﻿^The index ranges from 0 (complete integration) to 1 (complete segregation)

^b﻿^Proportion of people living under poverty line

**Table 4:** Continuous response linear mixed model coefficients for the complete model with isolation index as segregation variable

	**Females**				**Males**			
	**Brazil**	**Chile**	**Colombia**	**Mexico**	**Brazil**	**Chile**	**Colombia**	**Mexico**
**Fixed effects**	Coef (95%CI)	Coef (95%CI)	Coef (95%CI)	Coef (95%CI)	Coef (95%CI)	Coef (95%CI)	Coef (95%CI)	Coef (95%CI)
(Intercept)	25.9 (24.9—27.0)	26.7 (24.8—28.7)	25.3 (23.7—26.9)	28.6 (27.5—29.7)	25.2 (24.3—26.1)	25.2 (23.4—27.0)	24.0 (22.3—25.8)	26.2 (25.1—27.3)
City level								
Isolation^a^﻿	0.9 (-4.0—5.8)	7.8 (-4.0—19.7)	**-7.9 (-14.1—-1.7)**	5.9 (-1.5—13.4)	-3.3 (-7.4—0.7)	6.5 (-2.0—15.0)	-4.5 (-10.8—1.8)	3.7 (-3.6—11.0)
Poverty^b^	-0.5 (-5.3—4.4)	-7.6 (-23.2—7.9)	5.6 (2.1—9.0)	-4.1 (-6.4—-1.8)	3.0 (-1.0—7.0)	-2.4 (-12.9—8.1)	2.4 (-1.2—6.0)	-1.9 (-4.1—0.4)
Individual level								
Age[18,30]	Ref	Ref	Ref	Ref	Ref	Ref	Ref	Ref
Age(31,44]	1.9 (1.7—2.1)	1.5 (0.8—2.2)	2.1 (1.6—2.6)	2.7 (2.5—3.0)	1.9 (1.7—2.1)	1.6 (0.9—2.3)	2.2 (1.6—2.7)	2.3 (2.1—2.6)
Age(44,57]	2.4 (2.2—2.7)	2.3 (1.6—3.0)	4.0 (3.4—4.6)	3.5 (3.2—3.8)	2.2 (2.0—2.4)	2.2 (1.4—2.9)	1.7 (1.1—2.4)	2.4 (2.1—2.7)
Age(57,70]	2.4(2.2—2.7)	2.8 (2.0—3.6)	3.6 (2.8—4.3)	3.2 (2.8—3.6)	2.1 (1.9—2.4)	2.1 (1.3—3.0)	1.9 (1.1—2.6)	1.8 (1.5—2.2)
Education None	Ref	Ref	Ref	Ref	Ref	Ref	Ref	Ref
Education Primary	-0.2 (-0.4—0.1)	-0.2 (-1.1—0.8)	-0.4 (-1.0—0.2)	-0.2 (-0.5—0.1)	0.2 (-0.0—0.4)	0.2 (-1.1—1.4)	0.3 (-0.4—1.0)	0.5 (0.2—0.8)
Education Secondary	-1.0 (-1.2—-0.7)	-1.5 (-2.4—-0.5)	-0.8 (-1.5—-0.2)	-1.0 (-1.4—-0.7)	1.0 (0.8—1.9)	0.1 (-1.1—1.4)	0.5 (-0.2—1.2)	0.7 (0.4—1.1)
Education Tertiary	-1.9 (-2.1—-1.6)	-2.2 (-3.4—-1.0)	-1.0 (-1.9—-0.2)	-1.9 (-2.3—-1.4)	0.9 (0.7—1.2)	-0.8 (-2.2—0.7)	1.1 (0.2—2.1)	1.2 (0.8—1.7)
**Random effects**	Variance	Variance	Variance	Variance	Variance	Variance	Variance	Variance
City	0.186	0.320	0.152	0.456	0.113	0.000	0.147	0.412
Residuals	26.507	24.991	20.765	31.932	19.241	17.386	17.014	22.722
ICC	0.007	0.013	0.007	0.014	0.006	0.000	0.009	0.018

Results for intermediate models and models without stratification are available in Tables S2 and S3. In bold: confidence intervals that exclude 0, indicating statistically significant coefficients for the explanatory variable

*P*-values are available in Table S4

^a^The index ranges from 0 (complete integration) to 1 (complete segregation)

^b^Proportion of people living under poverty line

Of the 3 models for each segregation index, the complete model with all the covariates at the city and individual level was kept for interpretation, which corresponds to the one with the lowest AIC and BIC (Table S1). After adjusting for poverty, age and education, the relationship between BMI and segregation suggested effect modification by countries and between sexes, for both dissimilarity and isolation indices (Table S2). Also, in some countries, the direction and statistical significance of associations differed by sex at the city level, highlighting the case of Chile where women’s BMI increases when the dissimilarity index decreases, while the opposite occurs for men (Table 3, second line of coefficients). The interaction analyses showed no significant effect modification by poverty or by individual educational level.

High dissimilarity indicates an unequal distribution of disadvantaged groups in the urban space, which are underrepresented in some areas and overrepresented in others. In this study, this points to an under or overrepresentation of people with low SES in certain areas of the city. In Brazil, Colombia, and Mexico there is a negative relationship between dissimilarity and BMI for both men and women (Table 3). This means that BMI is lower in highly segregated cities, compared to those where disadvantaged and non-disadvantaged groups are more equally distributed in the urban space. However, although the coefficients were high, they were also imprecise, so that the confidence interval included the null in all cases except for Colombian men (-7.5 [95%CI -14.4 ‒ -0.5], *p*-value = 0.0358). In Chile, there was also a negative association between dissimilarity and BMI but only for men while the opposite occurs with women, indicating that in this country women have a higher BMI when the city is more segregated, while the opposite occurs with men; however, confidence intervals also included the null.

Segregation measured by the isolation index also showed an association with BMI that differed by country (Table 4). A high isolation index indicates the grouping of disadvantaged groups in such a way that the probability of them encountering the majority group gets diminished, which here means limited opportunities for interaction between people with low and high levels of education. In Chile and Mexico, isolation had a positive relationship with BMI for both sexes, indicating that people who live in highly isolated areas have a higher BMI in these countries. On the other hand, in Colombia the relationship was negative and statistically significant for women (5.6 [95%CI 2.1 ‒ 9.0], *p*-value = 0.0120), meaning that women who live in highly isolated areas have a lower BMI than those who live in areas with higher exposure to other social groups. In Brazil, the relationship was positive for women and negative for men.

## Discussion and conclusions

The goal of this study was to analyze the relationship between BMI and socio-economic residential segregation, measured from educational level or income. We expected to find that the most segregated cities would have more people with obesity and a correspondingly higher mean BMI. This relationship could be mediated by higher exposure to obesogenic environments in more impoverished and segregated neighborhoods [40, 43, 50]. Our results add substantial information to the very limited evidence on segregation and health across cities in Latin America.

We found that Chile, Mexico, and Brazil show that urban segregation measured by the isolation index is positively related to BMI, however, this was not the case in Colombia. We also found that in Brazil, Colombia, Chile, and Mexico there was an unexpected negative relationship between the urban dissimilarity segregation measure and BMI.

Sex was another variable that may have influenced the results. For example, the isolation index in Brazil presented an inverted relationship with BMI in men, while dissimilarity index effect in women in Chile went in the expected direction. These results are similar to those of other studies that show that gender disparities in the prevalence of overweight and obesity differ by setting, and are potentially influenced by factors like urbanization, physical activity, cultural values, and biological factors [24, 45, 52, 60]. Our findings on gender disparities are consistent with Robinovich et al. analysis for Chile in the isolation index, but not in the case of dissimilarity index. These effects might be explained by factors other than SES segregation, like psychosocial pathways and obesogenic environments, [29, 31, 40, 43, 45, 46, 52, 61] which we did not measure.

Many studies -particularly those based in the United States- have focused on race residential segregation, given the pervasive impact of structural racism in shaping living opportunities among Black people [18]. In Latin America, some authors have studied racial, ethnic, or SES residential segregation [20, 25]. For example, studies carried out in Mexico, Chile, Colombia and Brazil [21–24, 60], showed that there is significant SES residential segregation and that lower-income households tend to be more segregated than higher-income households. In addition, segregation has been increasing [22], large cities appear to be more segregated than small ones [21], and segregation is concentrated in the periphery of cities [23].

SES residential segregation in terms of education and income is often associated with marked differences across neighborhoods in social and physical environments, including limited availability and access to healthy foods, limited access to healthcare services, and limited economic and social mobility [39, 40, 52]. Even in the most segregated areas, the impact of segregation on obesity could be mitigated by changing the retail food environment in the areas experiencing higher obesity prevalence. However, the placement of grocery stores in food deserts may not necessarily lead to improvements in dietary behaviors or BMI. This increase in access will only be effective if coupled with policies and programs that make healthy choices affordable and train residents on how to prepare healthy meals, indicating that SES factors are also very important in dietary behaviors [41, 43]. Goodman concludes that when segregation is measured as exposure, the food environment likely mediates the relationship between segregation and BMI [31]. These could serve as explanations for our findings, many factors are mediators between segregation and BMI.

The results of this study support that individual and contextual factors are important in explaining individual BMI. The hypothesis was only validated for segregation measured by isolation index. The effect of residential segregation on BMI was observed to be influenced by sex at the individual level and as showed, has a different strength of association between countries. These differences could be mediated by other contextual variables not measured in this study.

The innovative nature of this inter-country study should be highlighted, given that there are no previous similar studies, and it is important for the debate on the contextual determinants of obesity like unhealthy food environments. However, some methodological limitations of our study -that are also described in the literature- may affect the interpretation of our results. Some of these difficulties result from data employed to determine a person's SES. Indeed, the effects of segregation on BMI were studied at the city level and not at the neighborhood level, although it is in neighborhoods where the greatest effects of segregation have been studied [40, 43, 50], and where these effects may have greater relevance for resident health behaviors. Despite this, our study manages to show a slight effect on cities (ICC in Tables 3 and 4) on their inhabitants, which would be expected to increase when analyzed at the neighborhood level. It could be explored by future work. Unlike previous studies of urban segregation at a neighborhood level [17, 19, 24, 62], this research employs a city-level approach to assess urban segregation. This is due to the small sample sizes of the national surveys at the neighborhood level, which impede the estimation of mean BMI for small spatial units. For this reason, future inter-country studies should consider an analysis that makes it possible to compare the effects of urban segregation at neighborhood and city levels on BMI.

It is important to identify environmental factors behind the inequalities affecting people's health. In Latin America, according to the World Health Organization, 25% of the disease burden is due to environmental influences, like urban design, health food availability, marketing exposure and others [63]. If we add inequalities produced by the social determinants of health, this percentage could increase considerably. An adjustment in the methodology for calculating the segregation indices or defining new indices that are adapted to the Latin American reality could be advantageous, particularly considering that in Latin America about 80% of the population lives in urban areas and these areas are experiencing a progressive increase in non-communicable diseases [39]. Identifying inequalities and comparing their magnitude both between countries and within the same country can contribute to developing public urban development policies with a health and quality of life perspective.

We conclude that the effect of socioeconomic segregation of Latin American cities on the obesity of individuals in those cities shows that urban segregation as measured by the isolation index is positively related to BMI, except in some groups. Individual-level variables explain most of the variability in BMI and have a smaller but more consistent impact than city-level variables. City level must be considered in the explanation of the phenomenon because the individual level is not enough to explain the correlation between people living in the same city. Further studies are needed to establish other factors that may be involved in the process, such as modifiable environmental characteristics that could be the target of future public policies.

### Supplementary Information

Supplementary Material 1.

Supplementary Material 2.

## Data Availability

The data that support the findings of this study are available from the SALURBAL project but restrictions apply to the availability of these data, which were used under license for the current study, and so are not publicly available. Data are however available from the authors upon reasonable request and with permission of the SALURBAL project. To learn more about SALURBAL’s dataset, visit https://drexel.edu/lac/ or contact the project at salurbal@drexel.edu.

## Abbreviations

AIC: Akaike Information Criterion
BIC: Bayesian Information Criterion
BMI: Body Mass Index
FAO: Food and Agriculture Organization of the United Nations
ICC: Intraclass Correlation Coefficients
PAHO: Pan American Health Organization
SALURBAL: Salud Urbana en America Latina/Urban Health in Latin America study
WHO: World Health Organization
