# Supplementary material for: Relationship between body mass index and residential segregation in large cities of Latin America
Source: BMC Public Health. 2024 Jun 22;24:1664. doi: 10.1186/s12889-024-19074-9 (PMC11193227; doi:10.1186/s12889-024-19074-9)
Supplement: Supplementary file 1 — Supplementary Material 1. [file 12889_2024_19074_MOESM1_ESM.pdf]

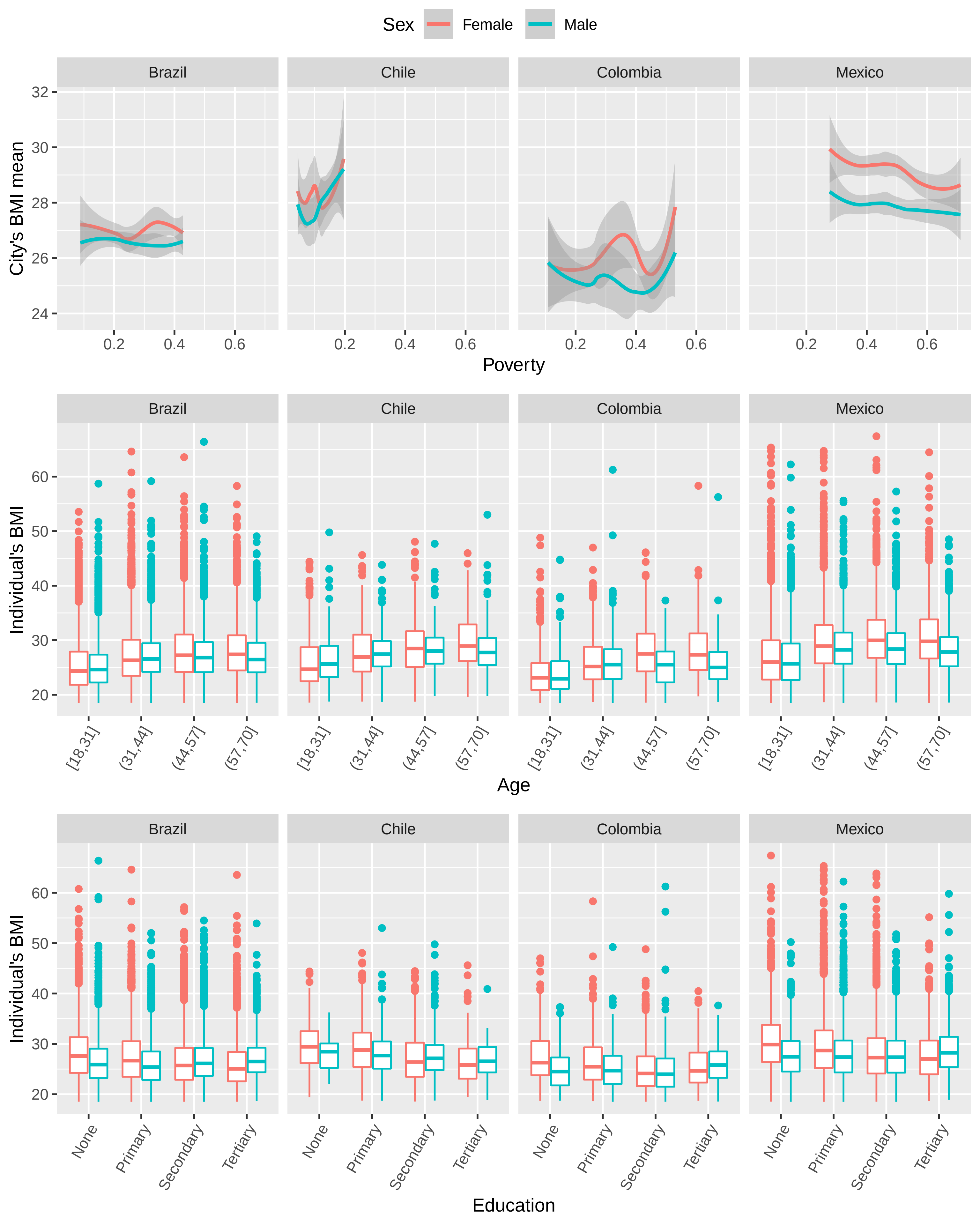

Sex
Female
Male
Brazil
Chile
Colombia
Mexico
City's BMI mean
Poverty
Individual's BMI
Age
[18,31]
(31,44]
(44,57]
(57,70]
Education
None
Primary
Secondary
Tertiary

Supplementary Figure 1: Body mass index distribution by sex and country for the adjustment variables in the study.

Upper panel contains city's mean BMI by the proportion of people under the poverty line in such city. Lines represent loess smoothers of the relationship between poverty and cities mean BMI, with 95%CI gray bands. Middle and bottom panels contain individual's BMI distribution by age group and by educational level, respectively. The lower and upper hinges correspond to the first and third quartiles; the upper whisker extends from the hinge to the largest value, no further than 1.5 times the inter-quartile range. Points beyond the end of the whiskers represent outliers.

BMI = Body Mass Index. Points represent cities and lines represent loess smoothers of the relationship between segregation indices and cities mean BMI. Red lines, points, and boxes represent females and blue lines, points, and boxes represent males.
